# Thaumatin-like proteins are differentially expressed and localized in phloem tissues of hybrid poplar

**DOI:** 10.1186/1471-2229-10-191

**Published:** 2010-08-26

**Authors:** Nicole J Dafoe, Brent E Gowen, C Peter Constabel

**Affiliations:** 1 Department of Biology, University of Victoria, Victoria, BC, Canada; 2 Centre for Forest Biology, University of Victoria, Victoria, BC, Canada; 3 Agricultural Research Service, US Department of Agriculture, Gainesville, FL, USA

## Abstract

**Background:**

Two thaumatin-like proteins (TLPs) were previously identified in phloem exudate of hybrid poplar (*Populus trichocarpa *× *P. deltoides) *using proteomics methods, and their sieve element localization confirmed by immunofluorescence. In the current study, we analyzed different tissues to further understand TLP expression and localization in poplar, and used immunogold labelling to determine intracellular localization.

**Results:**

Immunofluorescence using a TLP antiserum confirmed the presence of TLP in punctate, organelle-like structures within sieve elements. On western blots, the antiserum labeled two constitutively expressed proteins with distinct expression patterns. Immunogold labelling suggested that TLPs are associated with starch granules and starch-containing plastids in sieve elements and phloem parenchyma cells. In addition, the antiserum recognized TLPs in the inner cell wall and sieve plate region of sieve elements.

**Conclusions:**

TLP localization in poplar cells and tissues is complex. TLP1 is expressed predominantly in tissues with a prominent vascular system such as midveins, petioles and stems, whereas the second TLP is primarily expressed in starch-storing plastids found in young leaves and the shoot apex.

## Background

Two thaumatin-like proteins (TLPs) were recently identified in phloem exudate collected from hybrid poplar, *Populus trichocarpa *× *Populus deltoides *[1]. TLPs are named based on sequence similarity to the sweet tasting thaumatin protein from *Thaumatococcus daniellii *Benth [2] and belong to the PR-5 family of pathogenesis-related (PR) proteins [3]. The PR-5 family is one of 17 distinct PR protein families, and also includes the permeatins and osmotin. PR proteins typically accumulate to high levels following pathogen stress, but many are also inducible by other stress conditions or developmental cues. In some species TLPs are constitutively expressed in flowers and fruits, important reproductive organs that are susceptible to pathogen infection and it is hypothesized that they function as preformed defenses against infection [4-7]. TLPs have also been observed to be induced in response to wounding and insect feeding, specifically by phloem-feeding insects [8-12]. Currently relatively little is known about the function of TLPs in poplar, but transcriptomic experiments have shown that several TLPs are strongly upregulated by *Melampsora *infection [13,14], consistent with a function in pathogen defense. Some TLPs that are known to have antifungal activity act by permeabilizing fungal membranes [15]. Other TLPs appear to function by binding and hydrolyzing β-1,3-glucans [16-19], or inhibit fungal xylanases [20].

Our previous work showed that one of the TLPs in poplar phloem exudate, herein named TLP1, was wound-inducible, as it was present at higher levels in phloem exudate of plants whose leaves had been wounded 24 hours prior to collection [1]. Phloem exudate was collected from sieve tube elements, the specialized cells involved in long-distance phloem transport of angiosperms. At maturity, these cells no longer have a nucleus or functioning ribosomes. However, they retain their plasma membrane, endoplasmic reticulum, specialized plastids, and some mitochondria, although it is not known if these organelles are functional [21]. Sieve elements are known to contain many proteins [22], presumably transported to sieve elements from closely associated companion cells [23]. TLP genes are known to be expressed in phloem and TLP protein has been detected in phloem tissues in prior reports [24,25], but it had not been previously reported to be present in phloem sap. Therefore, we previously confirmed its presence within poplar stem sieve-tube elements by immunofluorescence [1]. In cross sections, the fluorescent label was clearly localized within sieve elements, and the label appeared to be punctate and associated with unidentified organelle-like structures [1].

Here, the expression and localization of TLPs in poplar sapling tissues was further characterized using the TLP1 antibody. Using immunofluorescence, the TLPs were observed to be constitutively expressed in hybrid poplar, specifically in phloem tissue. Immunogold labelling and electron microscopy was used to characterize the subcellular localization of TLPs within sieve elements, phloem fibres, and phloem parenchyma cells.

## Methods

### Plant materials

Poplar hybrid H11-11 (*Populus trichocarpa *× *P. deltoides*) saplings were propagated and grown in 2.5 L pots as described previously [26]. All plants were maintained in a greenhouse (16 h photoperiod) at the University of Victoria, British Columbia. The temperature was maintained at 25°C during the day and 18°C at night. Plants were watered daily with 0.1 g/L 20-20-20 PlantProd fertilizer (Plant Products, Brampton, ON, Canada).

### Tissue sampling and protein extraction

Samples from 3-month-old poplar saplings were collected from the shoot apex and petioles and blades corresponding to leaf plastochron index (LPI) 3-5, 9-11, and 15-17 [27]. Midveins were dissected and analyzed for LPI 9-11 and LPI 15-17, but insufficient quantities of protein for western blots were obtained from midveins of LPI 3-5. Bark (green stem tissue consisting of phloem, epidermal and cortical cells peeled from the wood or lignified secondary xylem) and wood samples were collected from LPI 9-11. Root samples were collected from areas of new root growth (young root) and areas near the base of the stem (old root). Plant tissue was frozen and ground in liquid N_2 _in a pre-cooled mortar and pestle. Total soluble proteins were extracted using sodium phosphate buffer (100 mM NaPO_4_, pH 7.0 containing 0.1% (v/v) Triton X-100, and 2% (v/v) β-mercaptoethanol) as previously described [28]. Phloem exudate was collected using an ethylenediamine tetraacetic acid (EDTA) method as previously described [1]. Tissue protein extracts and phloem exudate samples were precipitated with 2 vol cold acetone for at least one hour at -20°C. After 15 min of centrifugation at 16,000 × g at 4°C, the resulting pellet was dried and resuspended in Laemmli buffer [29] and quantified using the RC DC protein assay (BioRad, Hercules, CA, USA).

### Gel electrophoresis and immunoblotting

For immunoblotting, proteins (10 μg) were first separated with 15% polyacrylamide gels using a constant voltage (100 V) in a Mini PROTEAN II system (BioRad, Hercules, CA, USA). After separation, the proteins were electro-transferred to polyvinylidene fluoride (PVDF) membrane (Pierce, Brockville, ON, Canada). To detect TLPs, blots were incubated with a polyclonal antibody generated against recombinant TLP1 protein [1] and developed with an alkaline phosphatase conjugated-secondary antibody and 5-bromo-4-chloro-3'-indoylphosphate p-toluidine salt (BCIP) and nitro-blue tetrazolium chloride (NBT) as substrates. The specificity of the TLP1 antiserum was tested by labelling blots with preimmune serum or pre-adsorbed TLP1 antiserum. To pre-adsorb antiserum, 30 μL of TLP1 antiserum (total protein 1200 μg) was diluted 1:5 with deionized H_2_O and then incubated with an equal quantity of denatured recombinant TLP1 protein for 24 h at 4°C with constant shaking.

### Light microscopy and immunofluorescence

Petioles and midveins corresponding to LPI 3 and LPI 11 and stem segments near LPI 11 were excised, then fixed and embedded in BMM resin mixture (4 parts n-butyl methacrylate to 1 part methyl methacrylate) as previously described [30]. Cross sections of each tissue (6 μm thick) were prepared and labeled with the TLP1 antiserum as previously described [1] and the antibody was detected with a FITC AffiniPure goat anti-rabbit IgG (Jackson Immunoresearch Inc., West Grove, PA, USA). Controls included omitting the primary antibody or incubating sections with preimmune serum. Fluorescent labelling was visualized using a Zeiss Universal epifluorescence microscope equipped with a digital camera and a fluorescein isothiocyanate filter (excitation at 495 nm and emission at 519 nm).

### Electron microscopy

Stem sections near LPI 11 were collected and immediately placed in freshly prepared fixative buffer (sodium cacodylate buffer containing 3% (v/v) glutaraldehyde and 3% (v/v) formaldehyde [31]) for 1.5 h at room temperature with constant rotation. Samples were washed thoroughly with fixative buffer prior to dehydration with a graded ethanol series (50, 70, 80, 90, 95, and 2 × 100% (v/v) ethanol for 10 min each). After dehydration, samples were infiltrated with a mixture of ethanol and LR White (Electron Microscopy Sciences, Hatfield, PA, USA) at ratios of 1:1 and 1:3 for 30 min each, followed by two incubations with pure LR White for 30 min each. Samples were placed into individual gelatin capsules and then polymerized at 50°C for 24 h. Ultrathin sections were cut and mounted on formvar/carbon coated nickel grids (Electron Microscopy Sciences). Sections were first pre-treated with saturated sodium metaperiodate for 10 min and then incubated with 1% (w/v) ovalbumin (Sigma-Aldrich, Oakville, ONT, Canada) in phosphate buffered saline (PBS) for 10 min prior to labelling with TLP1 antiserum for 1 h. Sections were then washed three times, 5 min each, with PBS/ovalbumin and incubated with the secondary antibody (12 nm Colloidal Gold-AffiniPure Goat Anti-rabbit IgG (H + L), Jackson Immunoresearch Inc.) for 1 h. Sections were washed three times as described above and then washed four times for 1 min with deionized H_2_O. Prior to viewing, sections were stained with 5% (w/v) uranyl acetate in 50% (v/v) ethanol for 10 min, washed four times with deionized H_2_O, incubated with 5% (w/v) lead citrate for 1 min and washed an additional four times with deionized H_2_O. Sections were viewed with a Hitachi H-7000 transmission electron microscope and images were taken with a digital camera (Advanced Microscopy Technique Corp., Danvers, MA, USA). To verify the specificity of TLP1 antiserum, sections were also incubated with PBS/ovalbumin (no antibody), preimmune serum, or pre-adsorbed TLP1 antiserum. The density of gold labelling for each treatment was calculated by averaging the number of gold particles/μm^2 ^from five replicate areas in three separate stem tissue samples.

## Results

**Figure 1:**
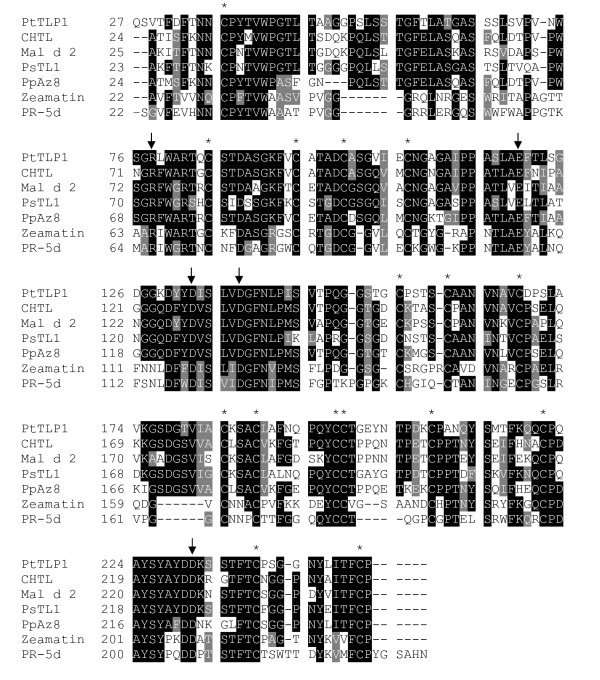
**Alignment of the mature, processed TLP1 protein sequence (JGI protein ID 828883) with the mature, processed sequences of previously characterized TLPs**. Sequences were retrieved from the National Center for Biotechnology Information database with the following accession numbers: CHTL (AAB38064), Mal d 2 (AAX19846), PsTL1 (BAA28872), PpAz8 (AAM00215), Zeamatin (P33679), and PR-5d (P258871). Black boxes indicate areas of identity and grey boxes represent amino acid changes that are conserved. Asterisks (*) indicate the position of conserved cysteine residues and arrows mark the position of the residues that contribute to the formation of an acidic cleft in TLPs.

The identification of a wound-induced TLP-like protein (JGI protein ID 828883), named TLP1, in poplar phloem exudate [1] prompted us to undertake a more detailed investigation of this protein. BLAST searches of protein sequences in the National Center for Biotechnology Information database (NCBI, http://blast.ncbi.nlm.nih.gov/Blast.cgi) identified TLPs from cherry fruit (CHTL), apple (Mal d 2), pear styles (PsTL1), and peach flowers (PpAz8) as the closest non-poplar homologs in GenBank. These proteins share greater than 60% amino acid identity and have several sequence features that are characteristic of TLPs. The five negatively charged amino acids that form an acidic cleft in PR-5 proteins [15,32-34] are present in TLP1 and the closely related TLP (Fig 1). Likewise, the TLP sequences all contain the 16 cysteine residues that are conserved in the subfamily of so-called large TLPs (Fig. 1). The predicted molecular weight of TLP1 protein is approximately 23 kDa, which is consistent with the molecular weight previously observed for TLP1 with two-dimensional (2-D) gel electrophoresis [1].

**Figure 2:**
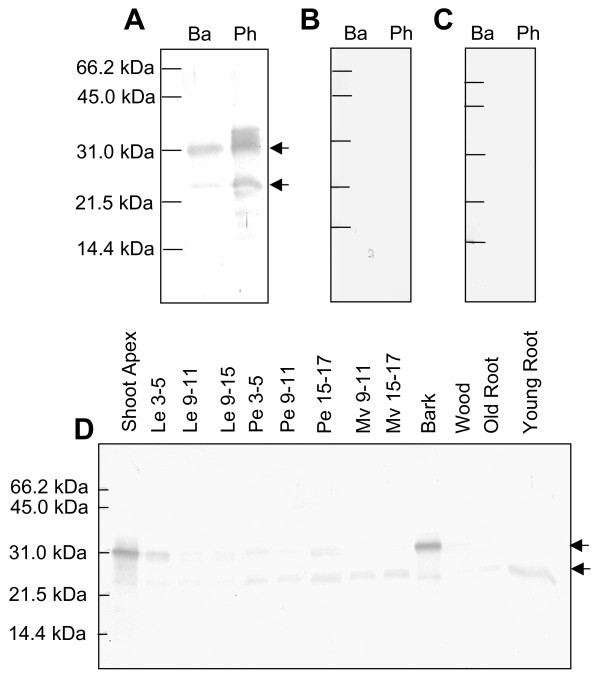
**Expression of TLP1 and closely related TLPs in *P. trichocarpa *× *P. deltoides *saplings**. Western blots of bark (Ba) and phloem exudate (Ph) proteins labeled with A) TLP1 antiserum, B) preimmune serum and C) pre-adsorbed TLP1 antiserum. D) Survey of poplar tissues by western blot labeled with TLP1 antiserum. Samples were collected from the apex, leaves (Le) and petioles (Pe) from positions LPI 3-5, 9-11, and 15-17, midveins (MV) from LPI 9-11 and 15-17, bark and wood collected from LPI 9-11 and old and young root samples. Arrows indicated the position of the 23 kDa and 31 kDa proteins detected by TLP1 antiserum.

As expected from its predicted and observed molecular weight, antibodies raised against recombinant TLP1 [1] detected a 23 kDa protein in poplar phloem exudate and in green stem tissue peeled from wood of poplar saplings, hereafter referred to as 'bark' (Fig. 2A). The antiserum also reacted with a 31 kDa protein band in both phloem and bark samples (Fig. 2A). No protein bands were labeled with preimmune serum (Fig. 2B) or pre-adsorbed TLP1 antiserum (Fig. 2C), indicating that the TLP1 antiserum was specific for both bands. To determine if this 31 kDa protein is a glycosylated form of TLP1, protein extracts were incubated with Concavalin A resin, a material that binds glycosylated proteins. Neither the 23 kDa nor the 31 kDa protein was retained by Concavalin A (data not shown), making it unlikely that the higher molecular weight protein is a glycosylated TLP1. Instead, these results suggest that this protein band corresponds to a closely related TLP protein. This hypothesis was supported by a parallel proteomic analysis of poplar phloem exudate, which identified a second TLP (JGI protein ID 583370), sharing 88% amino acid identity with TLP1. This second TLP migrated at 31 kDa on 2-D gels (Additional file 1), despite its predicted molecular weight of 24 kDa. The reason for the discrepancy between observed and predicted molecular weights is not known, but similar anomalies have been reported for other TLPs [35,36]. The second TLP shares long stretches of identical amino acids with TLP1, and it is thus likely that the TLP1 antibody can recognize both proteins.

Next, the TLP1 antibody was used to investigate TLP expression in poplar tissues. Both the 23 kDa and 31 kDa TLPs exhibited quite distinct tissue-specific expression profiles as visualized with western blots (Fig. 2). The 23 kDa protein was most abundant in petioles, midveins, bark, and roots, organs with well-developed vascular tissues. However, it was only weakly expressed in the shoot apex and young leaves (Fig. 2D). By contrast, the 31 kDa TLP showed the opposite pattern and was most abundant in these tissues but not significantly present in midveins or roots. Little or no signal was detected for either the 23 kDa or 31 kDa protein in older leaf and wood samples. These distinct expression profiles support the idea that the bands represent separate but closely related TLPs.

**Figure 3:**
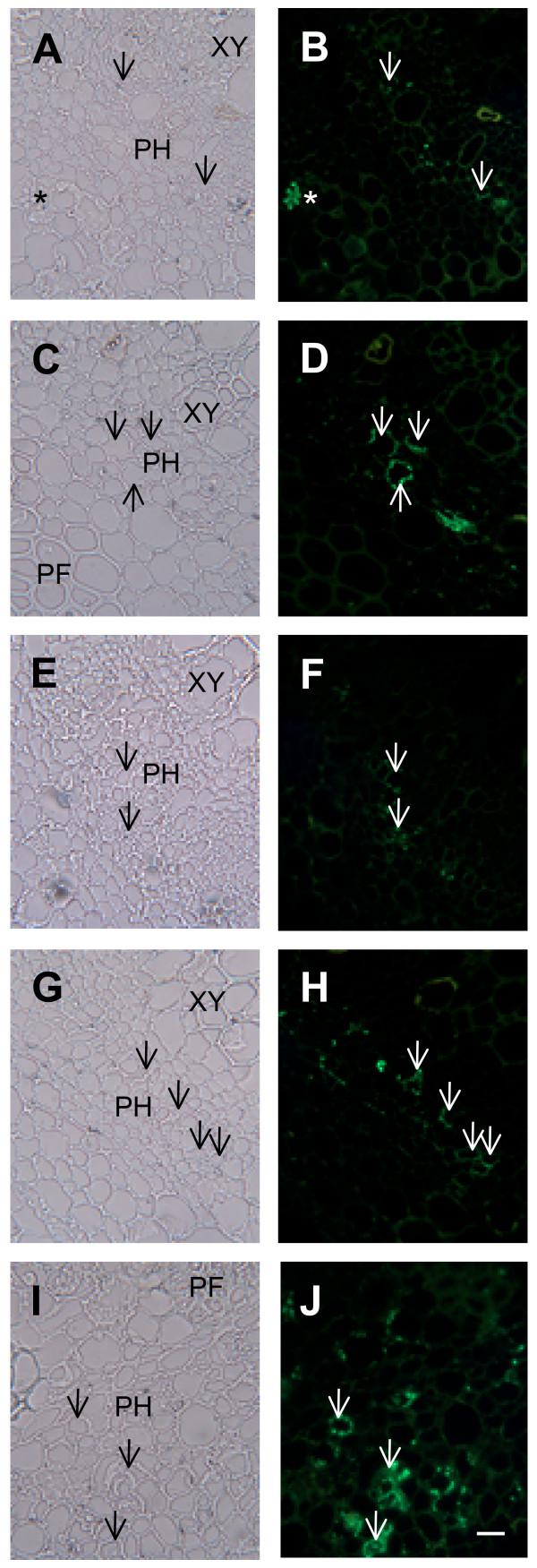
**Immunofluorescence of TLPs in cross-sections of phloem of diverse poplar tissues**. Panels A and B, young petiole sections (LPI 3); panels C and D, older petioles (LPI 11); panels E and F, young midvein (LPI 3); panels G and H, old midvein (LPI 11) panels I and J, stem (LPI 11) cross-sections, respectively, labeled with TLP1 antiserum. Panels A, C, E, G and I show bright field images, and panels B, D, F, H and J show the corresponding immunofluorescent images. PH, phloem; PF, phloem fibres; and XV, xylem vessels. Arrows indicate labeled sieve elements and asterisk indicates position of phloem parenchyma cell. Scale bar = 20 μm.

**Figure 4:**
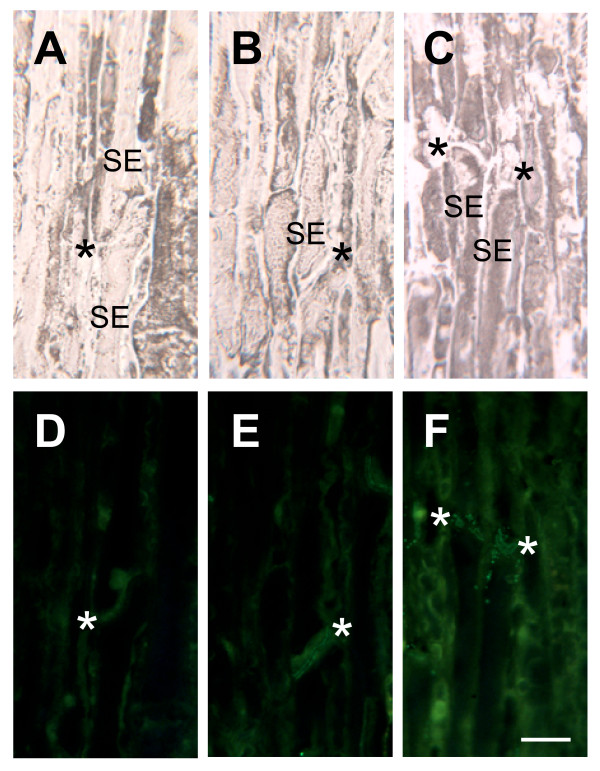
**Longitudinal sections of sieve elements (SE) in mature stem labeled with TLP1 antiserum**. Panels A-C show bright field images and panels D-F the corresponding immunofluorescent images. The stem section in panel D was treated without the primary antibody, E was treated with preimmune serum, and F was treated with TLP1 antiserum. Asterisks indicate position of sieve plate. Scale bar = 20 μm.

**Figure 5:**
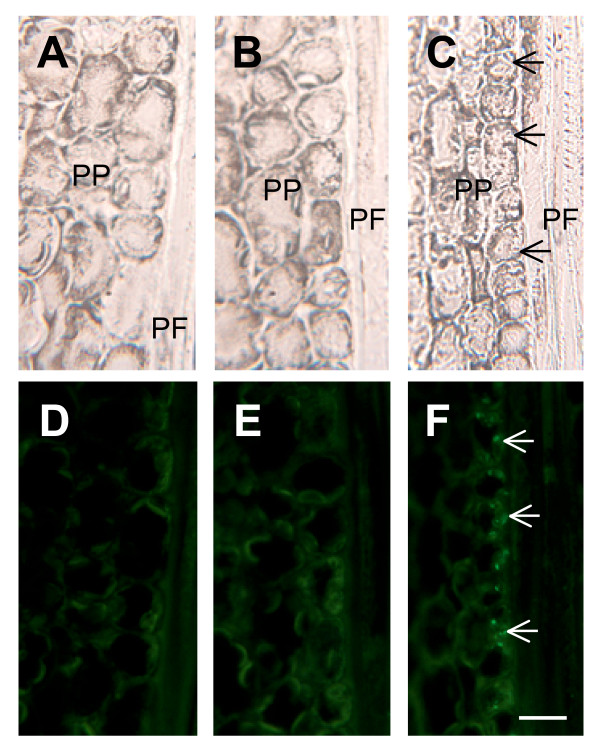
**Longitudinal sections of phloem parenchyma cells (PP) and phloem fibres (PF) in mature stem labeled with TLP1 antiserum**. Panels A-C show bright field images and panels D-F the corresponding immunofluorescent images. The stem section in panel D was treated without the primary antibody, E was treated with preimmune serum, and F was treated with TLP1 antiserum. Arrows indicate position of labeled phloem parenchyma cells. Scale bar = 20 μm.

Previous work indicated that TLPs are localized to punctate structures inside sieve elements within mature stem tissue. A phloem localization was expected since TLP1 was first identified in phloem exudate [1], but the punctate labelling was surprising. A similar punctate pattern was observed with immunofluorescence in petioles and midvein sections (Fig. 3). In general, this labelling was less intense when compared to the labelling observed in mature stem tissue, consistent with the western blot analysis showing that TLPs were more abundant in bark tissue than midveins and petioles. In petioles and midveins, the fluorescent label was detected in phloem cells between xylem vessels and phloem fibres. This labelling was punctate and appeared to be intracellular, similar to that observed in sieve elements in stem sections (Fig. 3B, D, F, H, J; 4F). In stem and young petiole cross sections, punctate, intracellular labelling was also detected in a second phloem cell type, phloem parenchyma cells, which were generally found in the vascular area adjacent to thick-walled phloem fibres (Fig. 3B, 5F). Overall, the intracellular labelling observed with the TLP1 antiserum was specific; very weak non-punctate fluorescence was detected when sections were labeled with PBS/ovalbumin (no primary antibody) or with TLP preimmune serum (Fig. 4D, E; 5D, E).

In mature stem, petiole and midvein sections, some fluorescent label also appeared to be associated with the cell periphery or cell wall. Unlike labelling observed within sieve elements and phloem parenchyma cells, this labelling was not punctate. Rather, it appeared to outline isolated phloem cells (Fig. 3D, H, J) suggesting that TLPs are present in cell walls or in structures closely appressed against these. Analysis of longitudinal sections through mature stem tissues indicated that this putative cell wall labelling occurred mostly in sieve elements (Fig. 4F). It was particularly prominent in the sieve plate region of the cell walls; however we note that this region was also weakly labeled by the preimmune serum (Fig. 4E). Therefore, the strong labelling observed at the cell periphery in some cells of the mature stem cross-section may reflect cell wall labelling of sieve plate sections (Fig. 3J).

**Figure 6:**
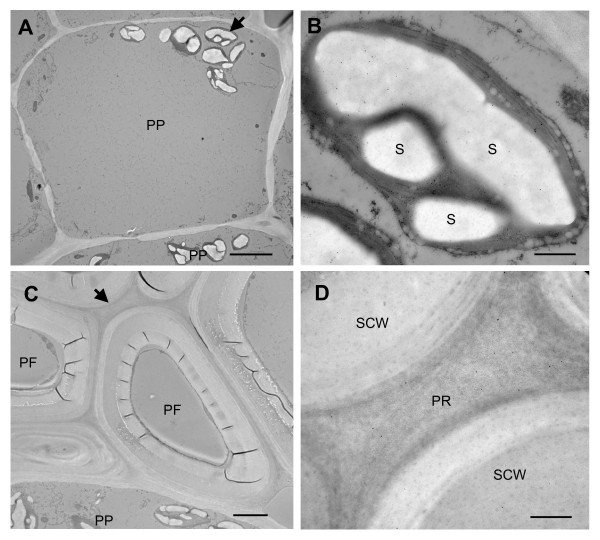
**Immunogold labelling of phloem cells with TLP1 antiserum**. A) Low magnification image of a phloem parenchyma cell (scale bar = 5 μm). B) High magnification image of starch-containing plastid showing TLP1 immunogold label (scale bar = 500 nm. C) Low magnification image of phloem fibre cells (scale bar = 2 μm). D) High magnification image of pectin-rich cell corner showing TLP1 label (scale bar = 500 nm). Arrows indicates positions of areas enlarged in panels B and D. PP, phloem parenchyma cell; S, starch; PR, pectin-rich region; PF, phloem fibre; SCW, secondary cell wall.

**Figure 7:**
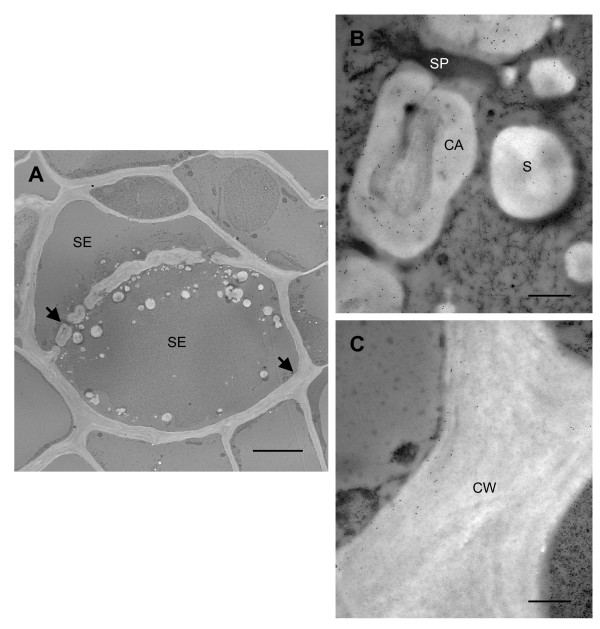
**Immunogold labelling of a sieve element (SE) in mature stem with TLP1 antiserum**. A) Low magnification image of sieve element. Scale bar = 5 μm. Arrows indicate position of sieve plate and cell wall that was enlarged in panels B and C, respectively. CA, callose; CW, cell wall; S, starch; SP, sieve pore. Scale bars = 500 nm.

To gain a deeper understanding of the localization patterns, we determined the subcellular localization of TLPs. Tissue sections from mature stems, which showed the highest level of expression of TLPs (Fig. 3), were analyzed with the TLP1 antiserum by immunogold electron microscopy. Within phloem parenchyma cells in sections of mature stem, we observed that starch-containing plastids were labeled with the TLP1 antiserum (Fig. 6B). Immunogold label was clearly associated with the starch granules as well as the stroma. Along phloem fibres, the gold label was concentrated in the pectin-rich cell corners between neighboring cells (Fig. 6C, D). By contrast, starch-like particles were again labeled in sieve elements (Fig. 7B), cells that were identified by their callose-lined sieve pores at the sieve plate (Fig. 7A). Numerous starch-like particles were detected inside sieve elements, but they were not surrounded by a membrane as seen in phloem parenchyma cells. Nevertheless, starch is synthesized within plastids, known to be present in sieve elements [37]. Staining with iodine confirmed that the particles found in the sieve elements are starch (data not shown). Therefore, the punctate label observed earlier by immunofluorescence most likely corresponds to labeled starch granules (Fig. 3J, 4F). As suggested by immunofluorescence (Fig. 3J, 4F), gold particles were also found in the inner layer of sieve cell walls and in the callose deposited in the sieve pores of sieve plates (Fig. 7B, C). The pattern of the gold particles on the inner layer of the cell wall was consistently seen in many sieve elements. No label was seen in the walls of other cells types, nor when antisera against other proteins were used (data not shown).

To confirm the specificity of immunogold labelling with TLP1 antiserum, gold labelling was quantified in stem sections labeled with PBS/ovalbumin (no primary antibody), preimmune serum (see Additional file 2), or TLP1 antiserum that had been pre-adsorbed with TLP1 protein in order to block TLP1 antibody binding. No label was detected when sections were labeled with PBS/ovalbumin, but some gold particles were detected with the preimmune serum and pre-adsorbed TLP1 antiserum in the cell wall and starch granules of sieve elements, and the starch-containing plastids in phloem parenchyma cells. Stronger labelling with the TLP1 antibody was observed in the callose found in the sieve plates. To determine the extent of background labelling in these cellular compartments, we quantified the average density of labelling for regions of interest in stem sections treated with TLP1 antiserum, preimmune serum, and pre-adsorbed TLP1 antiserum (Table 1). The density of labelling was also calculated for the cytosol of randomly chosen phloem cells (sieve elements, phloem parenchyma cells, and phloem fibres) as a background control for each treatment. With the exception of the cytosol, the labelling was consistently and significantly (Student's t-test, P < 0.05) higher in samples treated with TLP1 antiserum compared to either of the control treatments, indicating that the TLP1 antiserum is specifically labelling these regions in the various cell types. Surprisingly, immunogold labelling was also significantly higher in the callose region. However, strong labelling on callose was also observed with both the preimmune and pre-adsorbed antisera, making it difficult to interpret the callose results.

**Table 1:** Average density^a ^of preimmune serum, pre-adsorbed TLP1 antiserum, and TLP1 antiserum labelling.

	Preimmune Serum	Pre-adsorbed TLP1 antiserum	TLP1 antiserum
**Sieve Element**			
Cell Wall	0.50 (± 0.12)	1.37 (± 0.18)	5.35 (± 0.69)
Callose	22.37 (± 2.38)	9.99 (± 0.93)	35.10 (± 3.47)
Starch Granules	1.77 (± 0.36)	1.13 (± 0.40)	11.62 (± 1.92)
**Phloem Parenchyma**			
Plastids	1.46 (± 0.18)	0.89 (± 0.22)	4.46 (± 0.99)
**Phloem Fibres**			
Cell Corners	0.74 (± 0.13)	0.35 (±0.11)	2.41 (± 0.32)
**Cytosol**	0.14 (±0.04)	0.21 (±0.09)	0.24 (±0.08)

^a ^Average number of gold particles/μm2 + SE, n = 15

## Discussion

### Poplar contains closely-related TLPs

TLPs have been found in many different plant tissues and cell types, but their presence or role in phloem exudate and cells has not been studied in detail. Here we extend our investigations of TLPs in phloem tissues of hybrid poplar using an antibody produced against TLP1, a 23 kDa TLP previously identified in phloem exudate [1]. The antibody recognized a protein of the expected size in phloem exudate, bark, petiole and midvein, but also labeled a second band migrating at 31 kDa. Lack of binding to Concavalin A resin suggested this is not a glycosylated form of TLP1, and thus appears to represent a closely-related but distinct TLP. Consistent with this hypothesis, an abundant 31 kDa protein isolated from poplar phloem exudate was subsequently identified as a closely-related TLP. Sequence analysis indicated that the 31 kDa protein corresponds to JGI protein ID 583770 and shares greater than 80% sequence identity with TLP1. Thus, the TLP1 antibody could potentially cross-react with this TLP in poplar. A third TLP (JGI protein ID 669475) was also identified in poplar phloem exudate collected from mature stem tissue [1], but this protein is only 49% similar to TLP1 and is thus less likely to bind to the TLP1 antibody.

### Poplar TLP1-like proteins have a complex expression pattern and are present in plastids

A complex immunofluorescence and immunogold localization pattern was observed for TLP in phloem tissues, consistent with the idea that the antiserum detects at least two closely related TLP polypeptides. In stems, petioles, and midveins, the TLP1 antibody specifically labeled phloem cells, and this labelling was clearly intracellular and associated with starch and starch-containing plastids. In some cases the label was also detected in cell walls, sometimes within the same cells. For example, in sieve elements, TLPs were labeled on the inner cell wall as well as in starch granules. Although it is possible that a given TLP exhibits dual localization, we believe it is more likely that the TLP1 antiserum detects two closely related TLPs that are expressed in different subcellular locations. Based on the differential expression seen by western blots, the 23 kDa TLP1 is hypothesized to correspond to the label in the cell walls of sieve elements. This protein band was most abundant in organs that have well-developed vascular tissues and function in transport (petioles, midveins, stem bark, and roots). Interestingly, in sieve element cell walls, the label is restricted to the innermost layer (Fig. 7C). The significance of this distribution is not clear, but similar patterns cell wall layer-specific and cell type-dependent patterns labelling have been described [38].

In contrast, the 31 kDa TLP 583770 may be the TLP associated with plastids. On western blots, it was most prominent in bark and the apex, tissues in which immunofluorescence strongly labeled intracellular, organelle-like structures. In the bark, the TLP1 antiserum clearly labeled chloroplasts in phloem parenchyma cells, and starch granules, most likely originating from plastids (see below), in sieve elements. The 31 kDa TLP was also very abundant in the shoot apex, a tissue that does not have extensive vascular development. In this tissue, intracellular organelle-like structures, similar to the plastids observed in phloem parenchyma cells, were strongly labeled by immunofluorescence (data not shown). We note that while PR-5 and TLP proteins are most commonly isolated from cell walls and apoplastic fluids [3], they have also been described from plastids [39,40], including specific localization to starch granules in tomato chloroplasts [41]. Furthermore, other PR proteins which have been typically associated with the apoplast have also been detected in plastids. For example, PR-1 and PR-2 were found in plastids within styles and leaves in barley [39,42]. More work will be required to unequivocally connect a specific TLP gene product with its cellular localization; nevertheless, the current data shows that at least one of the poplar TLPs is located intracellularly in plastids in phloem tissue.

The structures labeled in phloem parenchyma cells are clearly starch-containing plastids, and immunogold labelling was detected in the plastid stroma as well as on the starch grains. In sieve elements, label was associated with 'free' starch granules with no surrounding plastids. Since starch is only synthesized in plastids, it is likely the starch granules we observed in sieve elements would normally be found in these organelles. Sieve elements contain two types of plastids, S- type plastids which contain only starch, and P- type plastids that contain protein, but may also include starch [37]. These plastids have been shown to be extremely sensitive to damage; they rapidly rupture after wounding events that result in a loss of turgor pressure [43,44]. Upon rupturing, the plastid membrane remains attached to the plasma membrane and its contents, including starch granules, are released [43,44]. This likely occurred to the plastids in our tissue samples as a result of the damage incurred during sample preparation. It has been hypothesized that these specialized plastids and their contents may contribute to the plugging of sieve pores after being released [21,44]. In light of the defensive potential of TLP and other PR-5 proteins, this is an attractive hypothesis.

### Phloem TLPs and potential roles in plant defense

TLP1 was first identified in phloem exudate 24 hours after wounding of leaves, suggesting that it may have a role in plant defense [1]. Other TLPs have been also shown to be induced in response to wounding and methyl jasmonate [11,12]. Given that some TLPs and PR-5 proteins have antifungal [16,45,46], and anti-insect activity [47], the function of the phloem-localized TLPs may be in defense against invaders that have breached the phloem. The specific localization of poplar TLPs in sieve elements may indicate a defensive role against phloem-feeding insects, since TLPs and other PR-5 genes are known to be induced in leaves in response to various phloem-feeding insects [8-10]. The presence of a TLP within plastids of sieve elements may also reflect a specific sequestration of defense proteins that can be rapidly released when damage to the cell causes these specialized plastids to burst.

One can also envisage roles of phloem-localized TLPs in poplar in pathogen defense. The amino acid sequence of TLP1 has 61% identity with Mal d 2, a TLP identified in apple fruit with reported antifungal activity [45]. Previous work already showed that TLP1 and TLP 583370, like many TLPs [3], are upregulated in response to *Melampsora *infection [13]. The localization of poplar TLPs to the cell wall is consistent with the cell wall or apoplastic localization of pathogen-inducible TLPs [11,40,48-50]. In addition, there is evidence that plastid-localized TLPs may function in pathogen defense. Some pathogen-inducible TLPs have also been localized to plastids [39-41], for example, a TLP was detected in plastid-like structures in Douglas fir roots seven days after the roots were infected by the fungal pathogen, *Phellinus suphurascens *[40]. In tomato plants, a PR-5 protein was detected in plastids where it specifically accumulated in chloroplast starch granules when plants were manipulated to express systemic acquired resistance [41].

## Conclusions

In summary, TLPs were found to be present in poplar phloem, specifically in sieve elements, phloem parenchyma cells, and phloem fibres. Like many TLPs [3], poplar TLPs were detected in cell walls, but they were also detected within cells, associated with starch and starch-containing plastids. This pattern of localization and is consistent with a role as a preformed defense against phloem-feeding insects and pathogens.

## Abbreviations

TLP: thaumatin-like protein; PR: pathogenesis-related; LPI: leaf plastochron index; BCIP: 5-bromo-4-chloro-3'-indolyphosphate *p*-toluidine salt; NBT: nitro-blue tetrazolium chloride; 2-D: two-dimensional; JGI: Joint Genome Institute

## Authors' contributions

ND carried out the protein work, microscopy and immunolocalization, and drafted the manuscript. BG helped with both light and electron microscopy, and immunolabelling. CPC conceived and helped plan the experiments, and participated in writing and revising the manuscript. All authors have read and approved the final manuscript.

## Supplementary Material

Additional file 1 **A 2-D gel of poplar phloem exudate proteins (50 μg)**. This figure shows the 2-D electrophoretic analysis of hybrid poplar phloem exudate proteins. This experiment was carried out to identify the most abundant protein spots visible by silver staining. The 31 kDa TLP that was sequenced by LC-MS/MS is circled. Click here for file

Additional file 2 **Preimmune serum-treated sections of sieve element**. This micrograph shows a control experiment to demonstrate the specificity of the TLP1 antibody. Sections of hybrid phloem cells were treated as for immune serum, except that preimmune serum was used as the primary antiserum. No immunogold label was detected. Click here for file
