# Supplementary material for: Thaumatin-like proteins are differentially expressed and localized in phloem tissues of hybrid poplar
Source: BMC Plant Biol. 2010 Aug 26;10:191. doi: 10.1186/1471-2229-10-191 (PMC2956541; doi:10.1186/1471-2229-10-191)
Supplement: Additional file 1 — A 2-D gel of poplar phloem exudate proteins (50 μg). This figure shows the 2-D electrophoretic analysis of hybrid poplar phloem exudate proteins. This experiment was carried out to identify the most abundant protein spots visible by silver staining. The 31 kDa TLP that was sequenced by LC-MS/MS is circled. [file 1471-2229-10-191-S1.PPT]

## Slide 1
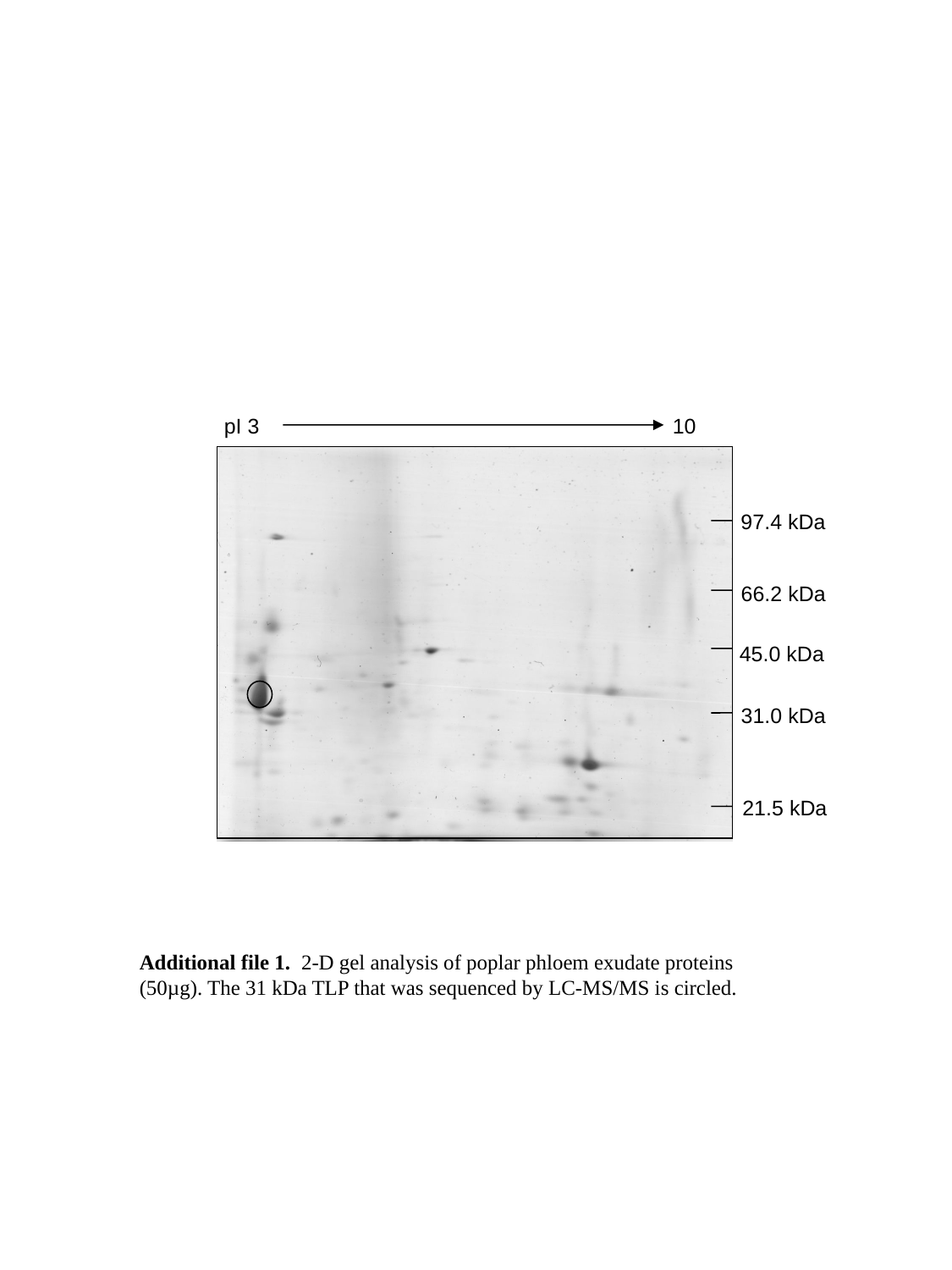

pI 3 10
97.4 kDa
66.2 kDa
45.0 kDa
31.0 kDa
21.5 kDa
Additional file 1. 2-D gel analysis of poplar phloem exudate proteins (50µg). The 31 kDa TLP that was sequenced by LC-MS/MS is circled.
