# Supplementary material for: Thaumatin-like proteins are differentially expressed and localized in phloem tissues of hybrid poplar
Source: BMC Plant Biol. 2010 Aug 26;10:191. doi: 10.1186/1471-2229-10-191 (PMC2956541; doi:10.1186/1471-2229-10-191)
Supplement: Additional file 2 — Preimmune serum-treated sections of sieve element. This micrograph shows a control experiment to demonstrate the specificity of the TLP1 antibody. Sections of hybrid phloem cells were treated as for immune serum, except that preimmune serum was used as the primary antiserum. No immunogold label was detected. [file 1471-2229-10-191-S2.PPT]

## Slide 1
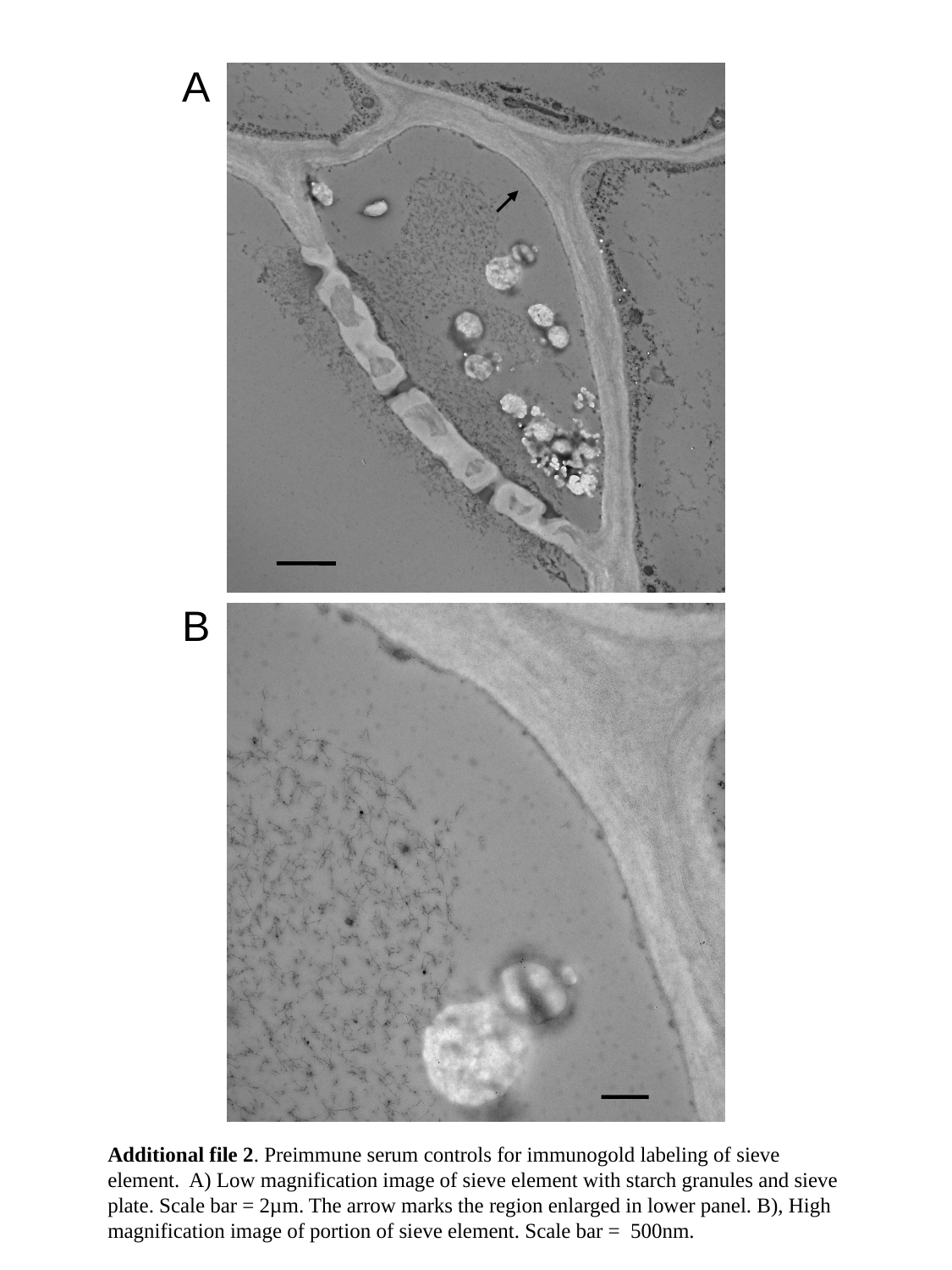

A
B
Additional file 2. Preimmune serum controls for immunogold labeling of sieve element. A) Low magnification image of sieve element with starch granules and sieve plate. Scale bar = 2µm. The arrow marks the region enlarged in lower panel. B), High magnification image of portion of sieve element. Scale bar = 500nm.
